# El derecho a mentir de los pacientes

**DOI:** 10.1016/j.aprim.2021.101967

**Published:** 2021-02-06

**Authors:** Jordi Delás, Rosario Salas, Ana Lozano, César Morcillo

**Affiliations:** a Servicio de Medicina Interna, Hospital Universitari Sagrat Cor. Departament de Medicina, Universitat de Barcelona, Barcelona, España; b Hospital Digital Sanitas. Medicina Interna, Hospital Sanitas CIMA, Barcelona, España

*Sr. Editor:*

Los artículos se leen por diferentes razones. Una de ellas, e importante, es por el título. Almadana et al.^1^ nos ofrecen un gran título tras el interrogante y las 3 primeras palabras. ¿Mienten los pacientes? Luego lo acotan al tabaco con delicadas referencias como información errónea, discordancias en el reporte del paciente, discrepancia informativa o no haber sido veraz, al punto que la palabra mentira no sale en todo el artículo. Con lo que el propio texto contextualiza la licencia del título, con el lícito objetivo de captar —como ha sido en nuestro caso— el interés de los lectores.

En la misma revisión de la bibliografía utilizada, de 24 referencias, solo una usa la palabra mienten y dicha formulación puede haber influido la magnífica elección del título por los autores^2^.

Creemos pues que, en la práctica, para los autores la mentira no es un concepto determinante, y queda abierto el debate sobre el derecho que tiene el paciente a suministrar la información, cuándo y en la proporción que quiera. Otra potestad similar al derecho a no saber^3^.

El término mayormente utilizado de tasa de decepción^3^ puede introducir un juicio sobre las expectativas en el interrogatorio de los pacientes. Si se acepta el derecho del paciente a la gestión de la información que quiere suministrar, la decepción desaparece. Queda como un elemento más en la comunicación médico-paciente, en la que este ha de sentirse lo más cómodo posible y no deben haber decepciones anunciadas.

De hecho, en la búsqueda de las palabras vinculadas *deception-rates* en PubMed*.*gov aparecen solo 3 referencias, lo que puede incidir en que los diferentes grupos de autores no han recurrido a este término con mucha frecuencia^4^, ^5^, ^6^.

Dentro de la incerteza clínica hay que abrir un importante apartado en la veracidad durante la anamnesis. Si convertimos la anamnesis entrevista en un duro interrogatorio, va a ser más fácil que nos engañen. ¿Hacen falta pruebas para contrastar la información del paciente? Cooximetría, gammaGT, tóxicos en orina. Quizás sí y quizás no. Más importante es que el médico sopese que su paciente dice la verdad quizás sí o quizás no. Que hace ejercicio de una potestad, decir o no la verdad, toda la verdad y nada más que la verdad.

En algunos campos puede viajar la mentira en bandeja de plata. «No se repondrá la prescripción médica bajo ningún concepto». Bajo ningún concepto puede desencadenar conceptos inimaginables por ninguno. El artículo citado hace referencia a dependencias legales. En las dependencias no legales, determinadas normas, reglamentos inducen ya a mentir para evitar expulsiones o interrupción de tratamiento.

¿Mienten nuestros pacientes? Tal vez, pero no lo incrementemos con el fondo y la forma de nuestras preguntas.
